# Reduced expression of mitochondrial lactamases in myeloid cells might be protective for AD

**DOI:** 10.1002/alz70855_099086

**Published:** 2025-12-23

**Authors:** Carmen Romero Molina, Wen Yi See, Ruben Gomez‐Gutierrez, Tulsi Patel, Jiacheng Ma, Hayk Davtyan, Mathew Blurton‐Jones, Jim Ray, Edoardo Marcora, Alison M. Goate

**Affiliations:** ^1^ Ronald M. Loeb Center for Alzheimer's disease, New York City, NY, USA; ^2^ Icahn School of Medicine at Mount Sinai, New York City, NY, USA; ^3^ The University of Texas Health Science Center at Houston, Houston, TX, USA; ^4^ Ronald M. Loeb Center for Alzheimer's Disease, Icahn School of Medicine at Mount Sinai, New York, NY, USA; ^5^ Department of Genetics and Genomic Sciences, Icahn School of Medicine at Mount Sinai, New York, NY, USA; ^6^ The Neurodegeneration Consortium, UT MD Anderson Cancer Center, Houston, TX, USA; ^7^ University of California Irvine, Irvine, CA, USA; ^8^ Ronald M. Loeb Center for Alzheimer's disease, New York, NY, USA; ^9^ Icahn School of Medicine at Mount Sinai, New York, NY, USA

## Abstract

**Background:**

The relevance of immunometabolism in AD requires further exploration. Our analysis integrating AD genetics and myeloid cell genomics reported that lower LACTB and LACTB2 expression are protective for AD. LACTB is a mitochondrial serin protein that may influence mitochondrial bioenergetics. LACTB levels are associated with succinyl‐carnitine levels (Suhre et al., 2011), a metabolite that has been linked to AD risk (Panyard et al., 2023). LACTB2 is a metallo‐protease that modulates mitochondrial mRNA levels and mitophagy. We aim to validate the link between LACTB/B2 and AD risk and understand the underlying mechanisms.

**Method:**

We generated LACTB/B2 KD THP1 macrophages and LACTB/B2 KO (CRISPR‐edited) induced pluripotent cell (iPSC) (2 donors), which we differentiated into microglia (iMGLs). Functional experiments (migration, phagocytosis, mitochondrial respiration, lysosomal activity or lipid droplets) were performed. Collected samples were outsourced for bulk and single‐cell RNAseq, metabolomics and lipidomics. In addition, we xenotransplanted human control and LACTB KO iMGLs into the brain of WT and 5xFAD mice.

**Result:**

we characterized, for the first time, the role of LACTB/B2 in myeloid cells. We observed that LACTB expression is increased upon differentiation and stimulation. LACTB KD/KO led to an increase in succinyl‐carnitine. In LACTB/B2 KD/KO myeloid cells, transcriptomics revealed an increase in oxidative phosphorylation (validated by Seahorse experiments) and in the response to interferon. By single‐cell, we observed that the highest LACTB expression corresponds to the TNF iMGL cluster. Lipidomics reported a significant increase in ceramides, which may suggest lysosomal alterations, and a reduction in acyl glycerides, pointing towards changes in lipid droplet accumulation. The engraftment of WT and LACTB KO human microglia in the mouse brain was successful. By immunostaining, we observed a mild reduction in amyloid pathology and an increase in microglia activation in 5xFAD mice xenotransplanted with human LACTB KO microglia.

**Conclusion:**

LACTB may play a role in cell differentiation and response to stimuli in myeloid cells by modifying mitochondrial respiration and lipid metabolism. Unlike other AD risk genes, LACTB encodes an enzyme, reduced expression is protective, and succinyl‐carnitine can be used as a biomarker, which highlights it as a promising AD therapeutic target.

